# Shaping the Future of Science: COVID‐19 Highlighting the Importance of GeoHealth

**DOI:** 10.1029/2021GH000412

**Published:** 2021-05-24

**Authors:** Morgan E. Gorris, Susan C. Anenberg, Daniel L. Goldberg, Gaige Hunter Kerr, Jennifer D. Stowell, Daniel Tong, Benjamin F. Zaitchik

**Affiliations:** ^1^ Information Systems and Modeling Los Alamos National Laboratory Los Alamos NM USA; ^2^ Department of Environmental and Occupational Health Milken Institute School of Public Health George Washington University Washington DC USA; ^3^ Department of Environmental Health Boston University School of Public Health Boston MA USA; ^4^ Department of Atmospheric, Oceanic, & Earth Sciences George Mason University Fairfax VA USA; ^5^ Department of Earth and Planetary Sciences Johns Hopkins University Baltimore MD USA

**Keywords:** GeoHealth, COVID‐19, air quality, communication, environmental justice, data

## Abstract

From the heated debates over the airborne transmission of the novel coronavirus to the abrupt Earth system changes caused by the sudden lockdowns, the dire circumstances resulting from the coronavirus disease 2019 (COVID‐19) pandemic have brought the field of GeoHealth to the forefront of visibility in science and policy. The pandemic has inadvertently provided an opportunity to study how human response has impacted the Earth system, how the Earth system may impact the pandemic, and the capacity of GeoHealth to inform real‐time policy. The lessons learned throughout our responses to the COVID‐19 pandemic are shaping the future of GeoHealth.

## Key Points

- The COVID‐19 pandemic has brought the field of GeoHealth to the forefront of visibility, especially by informing disease mitigation policies
- The pandemic provides an opportunity to observe how changes in human behavior have impacted the Earth system
- The pandemic is helping GeoHealth identify areas of future growth in data availability, environmental justice, and scientific communication

## 1 Introduction

**Figure 1:**
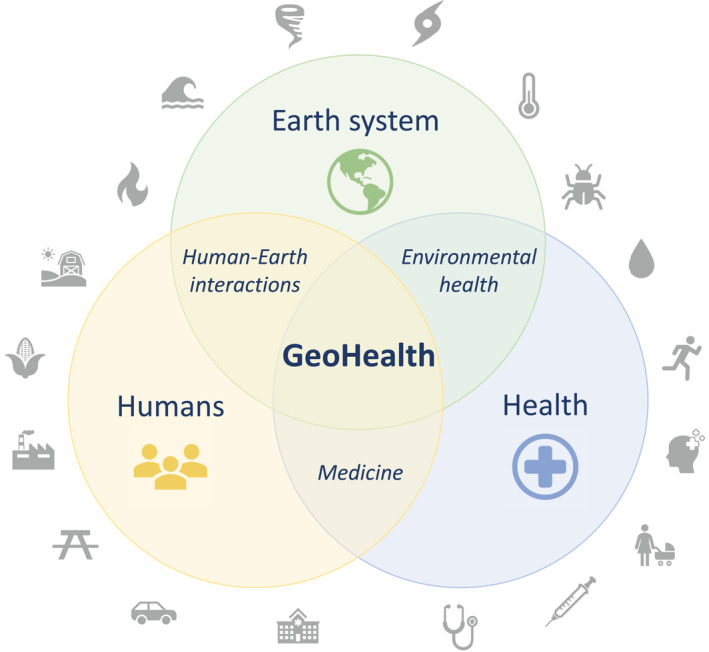
A Venn diagram of GeoHealth at the nexus of humans, health, and the Earth system.

The coronavirus disease 2019 (COVID‐19) pandemic is providing an unprecedented opportunity to observe how changes in human behavior during lockdown have impacted the Earth system, how aspects of the Earth system may affect COVID‐19 disease dynamics, and the role of geoscientists during the pandemic. The pandemic has highlighted the necessity of bridging the Earth system and human health through scientific research. Through these unfortunate and dire circumstances, GeoHealth has been brought to the forefront of visibility in the geosciences—especially throughout the American Geophysical Union Fall Meeting 2020 (AGU20) and in the public health policies designed to mitigate the spread of the disease. GeoHealth lies at the nexus of humans, health, and the Earth system (Figure 1), all of which are interconnected to the COVID‐19 pandemic.

Though the pandemic pushed many professional meetings, including AGU20, to a fully virtual platform, the scientific response and communication remained. AGU20 featured 47 sessions under the umbrella of COVID‐19, partitioned into three different themes: impact of COVID‐19 on the Earth system, impact of changes in the Earth system on COVID‐19, and science in the time of COVID‐19. Seven separate AGU sections featured sessions on COVID‐19 and there were 12 other supporting sessions formatted as innovative, union, plenary, and town halls.

The annual theme for AGU20 was “Shaping the Future of Science,” which was designed to focus on how the decisions we make now will affect the future. COVID‐19 and its connection to the Earth system has demonstrated how GeoHealth, a relatively new AGU section, is an imperative addition to the geoscience community. The COVID‐19 pandemic has pushed geoscientists to think outside the box and consider how our access to data and geospatial analytic methods can help the COVID‐19 response and inform both short‐ and long‐term Earth system responses (Diffenbaugh et al., 2020). However, COVID‐19 has hampered other scientific efforts, such as placing difficulty on geoscience fields that must travel to collect scientific samples (Scerri et al., 2020). In addition, not all scientists were affected the same: some have had more personal or family responsibilities that had to be prioritized, leading to decreased work productivity. Overall, research highlighting the COVID‐19 pandemic at AGU20 demonstrated the many ways GeoHealth is intertwined with other disciplines and an important, timely facet of geoscience.

## 2 The Impacts of COVID‐19 on the Earth System

The COVID‐19 pandemic has provided an unintended natural experiment to study how changes in human activity and emissions under lockdown affect the Earth system, both locally and globally. As exemplified across many talks at AGU20, one of the most salient impacts was the dramatic decrease in short‐lived atmospheric trace species. Primary species such as nitrogen dioxide (NO_2_) and aerosols measured from earth‐observing satellites and in situ monitors exhibited the most substantial decreases, while complex chemistry and competing influences from the biosphere and meteorology contributed to smaller, sometimes inconsistent, changes in secondary species (e.g., total fine particulate matter, or PM2.5, and ozone) and greenhouse gases (Archer et al., 2020; Goldberg et al., 2020; Le Quéré et al., 2020; Z. Liu et al., 2020; Siciliano et al., 2020; Zheng et al., 2020). Beyond satellite remote sensing, several academic and government institutions used chemical transport model simulations with adjusted emissions or business‐as‐usual simulations to identify air quality changes from the pandemic (Gaubert et al., 2021). Lockdown‐related emission changes could also feed back into the Earth system and influence hydrometeorological and temperature extremes due to microphysical and radiative forcing effects (Fuglestvedt et al., 2003; Gettelman et al., 2021). Beyond the atmosphere, the impacts of lockdown‐related changes in human activity extend to the hydrosphere (e.g., ocean acidification), biosphere (e.g., stressors on global fisheries), and lithosphere (e.g., reduced seismic activity). Research continues to explore the effects of changed human behavior on emissions through changes in energy use, human travel, and food security.

Although any changes in air quality or climate change are expected to be short‐lived or minimal as emissions return to prelockdown levels, the effects of the COVID‐19 pandemic on the Earth system have afforded the scientific community with lasting lessons. To assess COVID‐19‐related changes in geophysical parameters, physical measurements and models need to account for natural variability and complex Earth system processes. For example, attributing changing NO_2_ levels to the COVID‐19‐related anthropogenic activity change highlighted the importance of accounting for natural influences such as seasonal photochemistry and meteorological conditions (Gkatzelis et al., 2021; Goldberg et al., 2020). In addition, inconsistent changes in pollutants shed light on potential mitigation strategies aimed at reducing pollution and reveal persistent disparities in air pollution, exposure, and health outcomes. Some studies have found that changes in air quality were not only unequal spatially, but also varied among racial distribution and household income within cities, highlighting that pollution disparities persisted even despite the large‐scale decreases in traffic emissions (Kerr, Goldberg, et al., 2021). Lessons learned from this natural experiment can lead to more equitable environmental policies beneficial to human health, which is a primary focus for GeoHealth research.

## 3 The Impacts of the Earth System of COVID‐19

Apart from changes in human behavior during the COVID‐19 pandemic having an effect on the Earth system, the Earth system may also affect the pandemic. One of the largest areas of uncertainty in this regard is whether climate conditions will foster a seasonality in the transmission of COVID‐19, similar to influenza (Carlson et al., 2020; Kerr, Badr, et al., 2021; Kissler et al., 2020). Several talks at AGU20 explored the effects of environmental variables like temperature, humidity, aerosol settling time, and UV radiation on COVID‐19 dynamics. These environmental factors may affect the COVID‐19 transmission rate directly, through viral viability or human immune response, or indirectly by affecting human behavior (Kissler et al., 2020). Identifying patterns between climate conditions and COVID‐19 cases can shed light on environmental factors important for assessing disease risk. Further research to understand the relationships between environmental factors and COVID‐19 may inform more accurate forecasts of COVID‐19 and provide a knowledge basis for future emerging infectious diseases.

Major environmental disasters throughout the COVID‐19 pandemic have posed a complicated risk in disaster response by presenting competing priorities and co‐stressors in regard to human health and safety (Pei et al., 2020). Social distancing as a safety measure for COVID‐19 required agencies to reconsider evacuation plans and shelter use; for example, one agency created a framework to test the negative health outcomes from the compounding hazard of the pandemic and a tsunami, comparing the outcomes from enforcing different evacuation scenarios (Fry & Kong, 2020). Research across other natural disasters at AGU20 included the unprecedented wildfire seasons in Australia and the United States, the record‐breaking Atlantic hurricane season, the deadly volcanic eruption in the Philippines, earthquakes, and flash floods—events that all coincided with a pandemic. Continued research through the lens of GeoHealth can help resolve the negative health impacts related to each disaster versus outcomes from the pandemic, so that disaster response policies in the future can be better equipped to deal with multiple health hazards.

Knowledge from Earth science has played an important role in shaping public health policies to mitigate the global spread of COVID‐19. At the beginning of the COVID‐19 pandemic, public health agencies, including the World Health Organization, issued instructions on handwashing and social distancing to avoid infection, but messaging lacked agreement upon the role of airborne virus transmission and hence a mandate on wearing masks. Prior studies showed that speech droplets can be suspended in the air and the virus that causes COVID‐19 (SARS‐CoV‐2) may remain infectious for hours in the environment (Tang et al., 2020 and references therein). New findings reported at the AGU20 further provided direct evidence of the correlation between daily infections and speech aerosols (Gu et al., 2020). While airborne spread of COVID‐19 was not recognized as a primary route of exposure by many countries, geoscientists, based on the observations that aerosolized droplets can remain infectious in indoor air and be easily inhaled deep into the lungs, advocated that measures designed to reduce aerosol transmission must be implemented, including universal masking (Prather et al., 2020). Such groundbreaking work, through cross‐disciplinary collaborations and communication during the pandemic, exemplifies the key role played by the GeoHealth community to shift the policy paradigm toward more effective disease control strategies.

## 4 Science in the Time of COVID‐19

Earth science analyses during the initial stages of the COVID‐19 lockdowns rapidly shifted to better understand how society's response to the pandemic affected the Earth system. Due to the global coverage and consistent measurements by individual instruments, the existing constellation of satellite remote sensing instruments allowed an unprecedented observational record that could be used to understand environmental changes in real time. Recognizing the power of space‐based observations during this natural experiment, the European Space Agency, the Japan Aerospace Exploration Agency, and the US National Aeronautics and Space Administration cooperatively launched the Earth Observation dashboard (https://eodashboard.org/) for data on environmental and economic indicators, including agriculture, air travel, and air pollution changes. As was also discussed at AGU20, these examples of rapid international collaboration and openness in data and methods came alongside challenges associated with rapidly moving science. Huge numbers of COVID‐19 papers appeared on preprint servers prior to review, including many addressing GeoHealth topics of lockdown impacts or the influence of climate on the disease. These unvetted preprints were sometimes taken up prematurely by the media and policy makers (Carlson et al., 2020), presenting the GeoHealth community with the challenge of encouraging rapid and efficient communication while limiting the spread of potentially misleading analyses (Zaitchik et al., 2020).

Coupled with unprecedented air quality surveillance from some of these tools, the scope of pandemic‐related human mobility changes provided a unique opportunity to develop new scientific approaches for understanding human influence on the environment. For example, images of improved visibility in Los Angeles, US and Delhi, India widely circulated on social media and in the mainstream media, often attributing the clearer air to the drop in human mobility despite the fact that seasonal cycles and weather also substantially influence air quality (Holcombe & O'Key, 2020; Plumer & Popovich, 2020). Scientists rapidly developed novel approaches to disentangle the effects of anthropogenic emission change from natural variability using a wide range of methods, including satellite remote sensing (Bauwens et al., 2020; Ding et al., 2020; Goldberg et al., 2020; F. Liu et al., 2020; Sathe et al., 2020), chemical transport modeling (Gaubert et al., 2021; Keller et al., 2021; Miyazaki et al., 2020; Wang et al., 2020), ground observations (Berman & Ebisu, 2020; Chen et al., 2020; Fu et al., 2020; Parker et al., 2020; Tanzer‐Gruener et al., 2020; Turner et al., 2020; Venter et al., 2020), and air monitoring studies from aircraft (Frost et al., 2020; Ren et al., 2020). These new approaches can be valuable for future explorations of how rapid changes in human activity and/or policy influence air quality, given historic challenges with attributing air quality change to specific policies. Often, these stark changes in air quality provided an opportunity for scientists in the GeoHealth community to publicly communicate how human activities affect the environment (Asmelash, 2021; Davenport, 2020).

Many forms of scientific research were interrupted during the pandemic. Field and laboratory work ground to a halt at the beginning of the pandemic and were still substantially restricted a year later (Scerri et al., 2020). With school closings and reduced childcare options, many scientists have had fewer hours for research, teaching, professional development, and other scientific endeavors. As a whole, women have been more negatively affected, exacerbating the gender imbalance in academia and scientific research more broadly (Bell & Fong, 2021). Universities are also experiencing substantial strains as they formulate protocols and processes to operate safely, with virtual classes, extensive virus testing and tracing, and look to creatively adapt to societal changes in the future (e.g., with new online degree offerings). AGU20 was a prime example of science reimagined for the time of COVID‐19: typically drawing nearly 30,000 attendees each year with hundreds of oral and poster sessions, the meeting was revamped and offered virtually for the first time. This and other virtual scientific meetings can serve as models for increasing participation and fostering more worldwide collaborations while also reducing greenhouse gas emissions associated with conference travel (Coroama et al., 2012).

## 5 COVID‐19 and GeoHealth Shaping the Future of Science

The response of the geoscience community to the COVID‐19 pandemic has emphasized the strong points and weaknesses within data availability, the ability of scientists to communicate during a global crisis, and equity in GeoHealth. A theme throughout many COVID‐19 AGU20 sessions was a discussion around the time scales and higher resolution data needed to robustly assess the impacts of COVID‐19 on the Earth system. Having higher resolution spatial data that can differentiate metropolitan areas from other areas available at subdaily time scales could help untangle some of the more nuanced impacts of the pandemic on the Earth system. Scientific response to the COVID‐19 pandemic necessitated several new data repository hubs so that geophysical data are more readily available for researchers outside of the geosciences. The pandemic is exemplifying the utility in using forecasting methods, creating projection assumptions, and quantifying uncertainty commonly used in weather and climate products to forecast the spread of COVID‐19 (Bertozzi et al., 2020). The push towards higher resolution data, more readily available data, and innovative applications of disease forecasting will continue to benefit the current pandemic response and future studies in GeoHealth.

Another key scientific skill the COVID‐19 pandemic has amplified is the importance of effective scientific communication. Broadly, the pandemic has shown that people will not adhere to scientific facts and may attempt to invalidate scientific evidence in order to promote a personal or political agenda (Kouzy et al., 2020). This has already challenged the GeoHealth community through climate change skepticism (Dunlap, 2013). Scientific societies, such as AGU, must step in to advocate for their science and stand for the integrity of their work. GeoHealth researchers and other geoscientists must continue to clearly communicate their science in a way that exemplifies the broader implications of their research so that people can use it effectively to make personal life choices. One way to promote an effective use of scientific research is to connect with local communities. GeoHealth researchers should work to build partnerships with community officials and other nonscientific institutions to identify the needs within the community and help provide tools to direct decision making. For the COVID‐19 pandemic, this may look like GeoHealth researchers identifying communities who are most at risk for the negative health outcomes of the pandemic based on scientific evidence and communicating this to local officials in order to create a response plan (e.g., Fattorini & Regoli, 2020). It could also look like identifying communities that were still experiencing a disproportionately higher level of pollution despite lockdowns. Effective partnerships and communication will ensure that the results from GeoHealth research, which intrinsically has direct societal implications, may be acted upon.

Another aspect of GeoHealth research that the COVID‐19 pandemic is continuing to highlight is equity and environmental justice. AGU is working to create a strategic plan to model a diverse, equitable community among our organization. GeoHealth, as a section, is taking strides to tackle these issues by creating a Diversity subcommittee, publishing and supporting our section's Diversity Statement, and developing a new GeoHealth steering committee composed of diverse experts in the field outside of the current executive committee. GeoHealth, as well as other geoscience disciplines, must continue to promote a diverse team of researchers, recognize the importance of cultural diversity, and foster mentoring and education in order to strengthen the impact of the science. Having a diverse scientific team increases productivity and innovation (Hong & Page, 2004) and may help address issues of environmental justice by dismantling barriers for people from diverse backgrounds to engage in science (Garibay, 2013; Jimenez et al., 2019). Throughout the GeoHealth sessions at AGU20 on COVID‐19, it was evident that the pandemic is disproportionately affecting marginalized communities; air pollution is still higher among these communities despite overall reduced emissions (Kerr, Goldberg, et al., 2021), healthcare resources are scarcer, and the negative health outcomes are greater (Tai et al., 2020). Issues of environmental justice must remain a priority area of research for the GeoHealth community to shape the future of science into one with health equity.

The scientific response during the COVID‐19 pandemic has brought the field of GeoHealth to the forefront of visibility in the geosciences. AGU20, especially in its virtual format, provided a platform for scientists worldwide to gather and share the latest information on COVID‐19 and the geosciences through the lens of GeoHealth. The numerous COVID‐19 sessions hosted across different AGU disciplines exemplified how human health, such as a pandemic, can impact many parts of the Earth system. The formation of GeoHealth as a new section in AGU is an example of how the geoscience community has already shaped the future of science. In response to the dire circumstances faced by the COVID‐19 pandemic, the lessons learned within the geoscience community now are sure to accelerate positive change, especially within GeoHealth.

## Conflict of Interest

The authors declare no conflicts of interest relevant to this study.

## Data Availability

No data were used for the creation of our manuscript.
